# The macro and micro of chromosome conformation capture

**DOI:** 10.1002/wdev.395

**Published:** 2020-09-28

**Authors:** Viraat Y. Goel, Anders S. Hansen

**Affiliations:** Department of Biological Engineering, Massachusetts Institute of Technology, Cambridge, Massachusetts

**Keywords:** 3C technologies, 3D genome, chromosome conformation capture, genomic organization, Hi-C, Micro-C, nuclear architecture

## Abstract

The 3D organization of the genome facilitates gene regulation, replication, and repair, making it a key feature of genomic function and one that remains to be properly understood. Over the past two decades, a variety of chromosome conformation capture (3C) methods have delineated genome folding from megabase-scale compartments and topologically associating domains (TADs) down to kilobase-scale enhancer-promoter interactions. Understanding thefunctional role of each layer of genome organization is a gateway to understanding cell state, development, and disease. Here, we discuss the evolution of 3C-based technologies for mapping 3D genome organization. We focus on genomics methods and provide a historical account of the development from 3C to Hi-C. We also discuss ChIP-based techniques that focus on 3D genome organization mediated by specific proteins, capture-based methods that focus on particular regions or regulatory elements, 3C-orthogonal methods that do not rely on restriction digestion and proximity ligation, and methods for mapping the DNA–RNA and RNA–RNA interactomes. We consider the biological discoveries that have come from these methods, examine the mechanistic contributions of CTCF, cohesin, and loop extrusion to genomic folding, and detail the 3D genome field’s current understanding of nuclear architecture. Finally, we give special consideration to Micro-C as an emerging frontier in chromosome conformation capture and discuss recent Micro-C findings uncovering fine-scale chromatin organization in unprecedented detail.

**This article is categorized under:**

Gene Expression and Transcriptional Hierarchies > Regulatory Mechanisms Gene Expression and Transcriptional Hierarchies > Gene Networks and Genomics

## 1 INTRODUCTION

For all of their complexity and rich diversity of constituent cellular phenotypes, multicellular organisms can be characterized by a common foundation—their genome. With all of our cells sharing the same genetic code, regulation of gene expression serves as the root of heterogeneity in cellular identity, response, and role. Given all of the information (form, function, development from a single cell, etc.) that must be encoded in the human genome, it is perhaps no surprise that the diploid human genome is very long, spanning 6 billion base pairs. Stretched end-to-end, the DNA in each diploid human somatic cell would measure roughly 2 m; however, a need for space-efficient storage of DNA results in its compaction by orders-of-magnitude to fit inside small nuclei less than 10 μm in diameter (Greeley, Crapo, & Vollmer, 1978; Piovesan et al., 2019). Despite this compaction, DNA must also be dynamically accessible to allow gene activation, regulation, and replication as the cell grows, divides, and responds to stimuli. These considerations define the two seemingly contradictory challenges of chromatin organization: packaging DNA so that it fits within the cell while retaining sufficient accessibility for processes necessary for cell functionality.

Looking for the forces managing the balance of packaging versus functional accessibility, researchers dove into an exploration of the linear genome in the 1970s. The recombinant DNA revolution heralded the development of new experimental techniques for molecular genetics (e.g., isolating genes for study), and genes were sequenced for the first time (Lis, 2019). By the 1980s and 90s, scientists had uncovered a myriad of factors involved with transcriptional initiation and regulation at regulatory motifs proximal to the gene of interest (Lambert et al., 2018; Roeder, 2019). As our understanding of the complexity of transcriptional regulation deepened, however, it became apparent that proximal regulatory elements are just one part of a wider regulatory landscape. The subject of the 3D genome and distal regulation of transcription began capturing greater interest as researchers identified regulatory elements termed enhancers thousands to millions of base pairs distal to their target genes (Spitz, 2016). For instance, a nuclear ligation assay developed in 1993 probed the rat *prolactin* gene and reported that the distal enhancer and proximal promoter regions are spatially juxtaposed, an interaction stimulated by estrogen ligand acting upon an estrogen receptor bound to the distal enhancer (Cullen, Kladde, & Seyfred, 1993; Gothard, Hibbard, & Seyfred, 1996). Given the central role that gene expression plays in cell phenotype and the onset of disease, unpacking the functional ramifications of these distal genetic interactions holds great promise for advancing our understanding of the genome and has thus become the impetus for the development of chromosome conformation capture technologies. In this review, we chronicle major developments in chromosome conformation capture technology and the biological insights their application has given us, with particular attention given to the recently developed Micro-C method.

## 2 THE DEVELOPMENT OF CHROMOSOME CONFORMATION CAPTURE TECHNOLOGIES

### 2.1 Chronology of key technologies and the features they detect

#### 3C, 4C, and 5C

**FIGURE 1:**
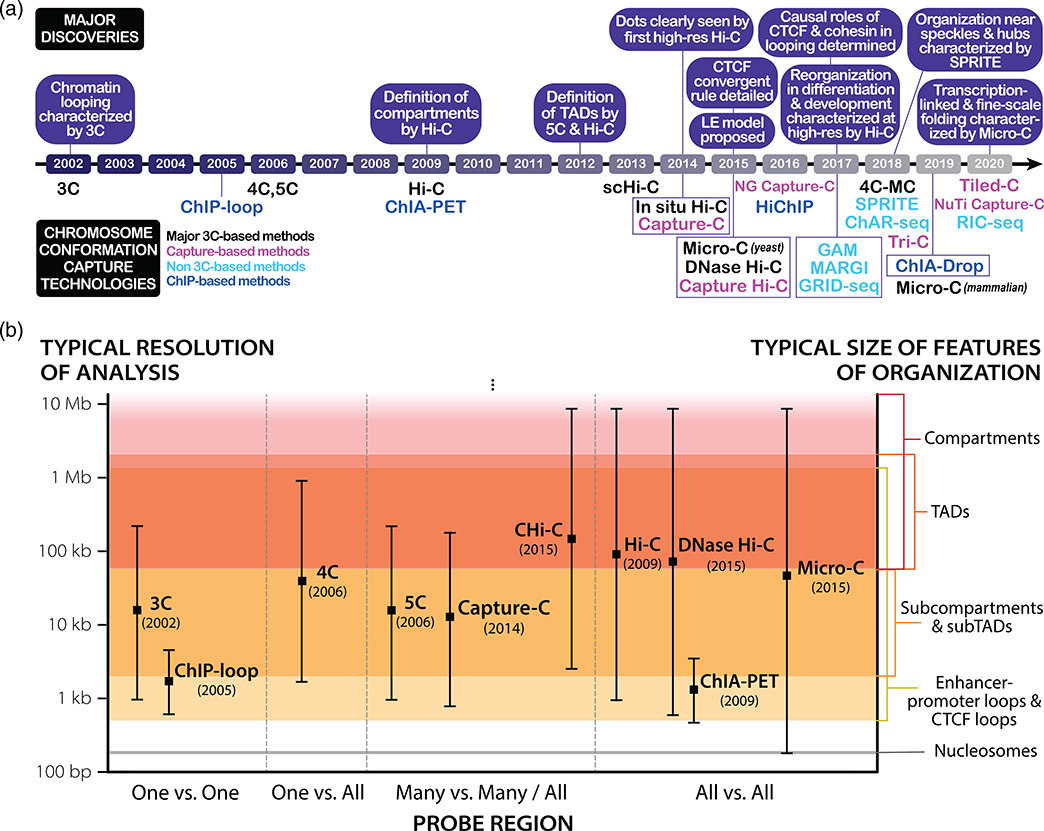
Timeline and comparison of major chromosome conformation capture techniques. (a) Chronological development of chromosome conformation capture technologies colored by type of method. Major observational, mechanistic, or biological discoveries are listed above the timeline. (b) Comparison of landmark 3C-based methods and the resolutions at which their datasets are typically analyzed. The typical resolution ranges for these technologies are historically grounded and may widen or shift with the inclusion of recent advances in methodology. Resolutions at which key features of chromatin organization typically manifest are shown on the right

Chromosome conformation capture technologies have primarily derived from the foundation laid by 3C (Chromosome Conformation Capture) in 2002 (Figure 1a; Dekker, Rippe, Dekker, & Kleckner, 2002). First developed in yeast (Dekker et al., 2002) and soon adapted for mammalian cells (Tolhuis, Palstra, Splinter, Grosveld, & de Laat, 2002), 3C is capable of probing pairwise interactions between specific genetic loci of interest and generating population-averaged contact frequency estimates between two chromosomal loci. Moreover, applying 3C to estimate the pairwise interaction frequencies of multiple loci enables the development of experimentally-constrained 3D polymer models of chromosomes (Dekker et al., 2002). The unique ability of 3C to precisely focus on specific loci and generate such models at relatively high resolution overcame some of the limitations of microscopic methods such as electron microscopy (which lacks locus specificity) and fluorescence in situ hybridization (FISH, which is a lower throughput technique) previously harnessed in probing nuclear architecture (Barutcu et al., 2016; Belmont, 2014; Sati & Cavalli, 2017). The steps at the heart of the 3C protocol—namely, chemical fixation and cross-linking of DNA, restriction enzyme (RE) digestion, proximity ligation, cross-linking reversal, and PCR amplification to generate the interactome library—allow 3C to achieve its specificity (by virtue of locus-specific amplification) and throughput (by virtue of its scalability to the whole-genome scale) and have since become a mainstay of the 3D genome field.

However, 3C only probes interactions between pairs of loci for which PCR primers have been designed, making it a low-throughput technique that is normally analyzed on gels or with RT-qPCR. This limitation as a “one versus one” method (Figure 1b) prompted the development of two higher-throughput derivatives, Circular Chromosome Conformation Capture or Chromosome Conformation Capture-on-Chip (4C) and Chromosome Conformation Capture Carbon Copy (5C), in 2006 (Dostie et al., 2006; Simonis et al., 2006; Z. Zhao et al., 2006). 4C modifies the 3C protocol by circularizing ligated fragments, allowing inverse PCR amplification that only requires primers for one of any two ligated fragments; thus, 4C is capable of mapping the interactions between a known locus of interest and the entire genome (a “one versus all” method, Figure 1b; Simonis et al., 2006; Z. Zhao et al., 2006). In its initial application, 4C examined the H19 imprinting control region and revealed that direct long-range interaction between methylated regions can serve as an epigenetic regulatory mechanism for transcription (Z. Zhao et al., 2006). By contrast, 5C amplifies select parts of a 3C library by using PCR primer pairs to focus on a region of interest for analysis via sequencing or microarrays (Dostie et al., 2006). Accordingly, 5C is able to probe pairwise interactions across a whole region of interest (a “many versus many” method, Figure 1b) and revealed looping interactions within the genome, affirming on a broader scale prior 3C studies on looping in the β-globin locus (Dostie et al., 2006; Tolhuis et al., 2002; Vakoc et al., 2005). Subsequently, researchers studying transcriptional regulation of the X-inactivation center in mouse embryonic stem cells (mESCs) using 5C and FISH discovered the presence of 200 kb–1 Mb sized self-interacting DNA regions they termed topologically associating domains (TADs; Nora et al., 2012). TADs define local regions of the genome that preferentially self-interact at a significantly increased frequency (typically ~2–3 fold greater) relative to regions outside of the TAD (Chang, Ghosh, & Noordermeer, 2020; Dixon et al., 2012; Nora et al., 2012; Rao et al., 2014). TADs are demarcated by clearly defined boundaries and can nest within compartments or within one another as smaller “subTADs” manifest within larger TADs. Despite the ubiquity of TADs as features of the 3D genome, it is important to note that the field currently lacks a unified definition for what constitutes a TAD and uncertainty remains in terms of nomenclature (Box 1; Beagan & Phillips-Cremins, 2020; Rowley & Corces, 2018). Despite their description less than a decade ago, TADs are now recognized as a hallmark of chromatin organization at the scale of tens of kilobases to megabases.

#### Hi-C

**FIGURE 2:**
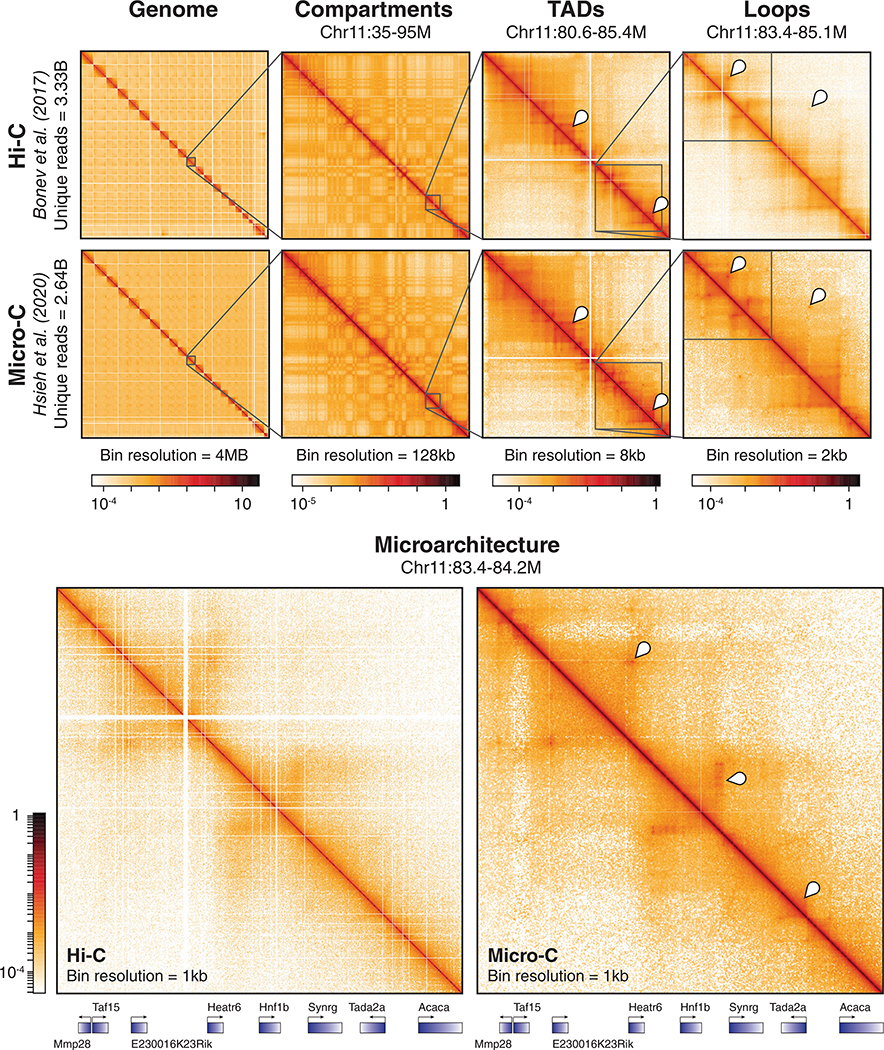
Micro-C captures finer-scale features of chromatin organization than Hi-C. (Top row) High-resolution Hi-C (Bonev et al., 2017) and Micro-C (Hsieh et al., 2020) datasets generated from wild-type mESCs are visually juxtaposed at various scales of chromosomal organization. The data is binned at different resolutions using HiGlass, with the visualization of any particular feature requiring bins of a finer resolution than the size of the feature. Contact heatmaps of the whole genome, compartments, topologically associating domains (TADs), and loops are shown. The checkerboard pattern in the second column of plots indicates separation into A/B compartments. The markers in the third column of plots indicate TADs, while the markers in the fourth column of plots indicate corner peaks or “dots” specifying loops. (Bottom row) Fine-scale resolution maps of the same Hi-C and Micro-C datasets. Markers identify microarchitecture, such as enhancer-promoter (E–P) or promoter-promoter (P–P) loops, stripes, and domains, visible in Micro-C but not discernable in Hi-C, and genes within the region are annotated below. This figure is inspired by fig. 1d and S1d in Hsieh et al. (2020)

Although 3C, 4C, and 5C allowed long-range DNA interactions to be studied, their reliance on researchers choosing target loci of interest prevented them from probing the whole genome in an unbiased manner. The advent of high-throughput chromosome conformation capture (Hi-C) in 2009 altered this paradigm (Lieberman-Aiden et al., 2009). A genome-wide adaptation of 3C, Hi-C utilizes biotinylation to enrich for proximity ligated contacts and modifies the library amplification process to utilize universal adapters and primers for high-throughput sequencing. Agnostic to the specific sequences being amplified, Hi-C can thus probe all genomic interactions in an unbiased “all versus all” approach (Figure 1b). The original Hi-C protocol, also referred to as dilution Hi-C, uses dilute proximity ligation conditions to minimize artifacts following nuclear lysis; this methodology was altered in the development of subsequent Hi-C derivatives. First tested in a human lymphoblastoid line, the initial application of low-resolution Hi-C (1 Mb-level resolution, achieved with 8.4 million reads) revealed the presence of preferentially self-interacting A and B compartments as a novel level of genome organization (Figure 2; Lieberman-Aiden et al., 2009). “A” compartments largely contain DNA classified as euchromatin that is more transcriptionally active, less densely packed, and localized away from the nuclear periphery, with the notable exception of nuclear pore complexes (Gozalo et al., 2020; Hildebrand & Dekker, 2020). By contrast, “B” compartments largely contain DNA classified as heterochromatin that is transcriptionally inactive, more densely packed, and localized near the nuclear periphery or the nucleolus (Hildebrand & Dekker, 2020). Manifesting genome-wide at the megabase scale and capable of checkering across chromosomes (unlike TADs), compartments exhibit a significantly higher frequency of long-range inter- and intrachromosomal interactions with DNA in the same compartment type compared to DNA in the alternative compartment type (Lieberman-Aiden et al., 2009) and correlate strongly with patterns in the timing of DNA replication (called replication timing domains) observed by BrdU labeling and Repli-seq (Hiratani et al., 2008; Rhind & Gilbert, 2013; P. A. Zhao, Sasaki, & Gilbert, 2020).

Equipped with a genome-wide method for conformation capture, researchers began exploring sub-megabase levels of nuclear architecture across the genome. The opening of this sub-megabase frontier was intimately linked to advances in DNA sequencing, with costs dropping faster than Moore’s law from 2008 onward due to the advent of next-generation sequencing (Heather & Chain, 2016; Wetterstrand, 2020). Innovation in sequencing was a necessary companion to Hi-C because, unlike nonpairwise genomic sequencing methods (e.g., RNA-seq, ChIP-seq) whose required sequencing reads for a resolution *n* scales linearly with genomic size, increasing the resolution of a genome-wide Hi-C pairwise contact matrix *n*-fold necessitates *n^2^* reads. Thus, sequencing costs serve as a limiting factor for the depth at which the interactome is captured.

Early Hi-C analysis of genomes in hESCs, mESCs, and differentiated cell types identified TADs as genome-wide features of mammalian nuclear architecture and reported that TADs are largely invariant between cell types, evolutionarily conserved, and separated by boundary regions enriched for factors and housekeeping genes of interest (Dixon et al., 2012; Phillips-Cremins et al., 2013). Subsequently, the first high-resolution contact maps generated by the application of in situ Hi-C in human and mouse cell lines revealed levels of genome organization as fine as the 1-kb scale from ~5 billion sequencing reads (Rao et al., 2014). Dots (corner peaks) were now clearly visible in Hi-C maps, and domains with clear corner peaks were termed loop domains (Figure 2). By comparing with ChIP-seq analyses, Rao et al. reported that 86% of these loop domains are bound by the CCCTC-binding factor (CTCF), 92% of which demarcate loop boundaries in a convergent orientation, and that 86–87% are bound by cohesin (a Structural Maintenance of Chromosomes, or SMC, complex) subunits RAD21 and SMC3 (Rao et al., 2014). Of the 2,854 loops identified as involving enhancers and promoters (E–P loops), 557 were cell-type-specific and also strongly correlated with cell-type-specific gene activation, thus ascribing a more definitively functional role to contact map features (Rao et al., 2014). Further probing the relationship between nuclear architecture, gene expression, and cell fate, the first high-resolution Hi-C analysis of development mapped 3D genome organization during mouse neural differentiation in vitro and in vivo (Bonev et al., 2017). Examination of the data revealed that transcriptional changes during differentiation are correlated with alterations in the strength of long-range interactions and the emergence of cell type-specific enhancer–promoter (E–P) contacts. Bonev et al. also found that such E–P interactions occur primarily within the same TAD and are generally established alongside gene expression, affirming similar findings of how TADs constrain enhancer activity (Symmons et al., 2014) and further connecting form and function. Critically, insights into the extensive genomic rewiring of structure during development underscored the dynamism of nuclear architecture and helped shift the field from a fairly cell-type invariant view of chromatin spatial organization toward a more cell-type-specific one (Beagan et al., 2017; Bonev et al., 2017; Pękowska et al., 2018). A comparison and timeline of key chromosome conformation capture methods are shown in Figure 1, and a visualization of Hi-C map features is shown in Figure 2.

### 2.2 Other 3C-derived technologies

As a rapidly blossoming field, nuclear architecture has witnessed an explosion in technology development as 3C-based methods have been modified to create a diverse array of derivatives. Many of these derivatives are designed to address the shortcomings and limitations of their parent technologies; others act to incorporate breakthroughs in adjacent fields. Some common modifications to parent protocols are reflected by a sampling of noteworthy 3C, 4C, and 5C derivatives. In an effort to bypass potential biases introduced by chemical cross-linking, intrinsic 3C (i3C), and 4C (i4C) forgo cross-linking and perform digestion and ligation in situ (discussed below); this not only reconstitutes known features of folding, but also improves the signal from more stable chromatin interactions (Brant et al., 2016). 4C-seq improves 4C’s resolution and reproducibility by introducing a second round of restriction digestion and ligation, and improves 4C’s throughput by incorporating adapters for NGS (van de Werken et al., 2012). The addition of unique molecular identifiers (UMIs) to 4C-seq in UMI-4C further refines the protocol, improving sensitivity, specificity, and multiplexing (Schwartzman et al., 2016). Finally, 5C-ID performs ligation-mediated amplification with a double alternating primer design and uses in situ digestion and ligation, resulting in reduced noise, improved sensitivity to loops, and fewer required input cells than native 5C (J. H. Kim et al., 2018). Major categories of 3C-based derivatives—namely, in situ and single-cell Hi-C, ChIP-based methods, and capture-based methods—are briefly discussed here.

#### In situ and single-cell Hi-C

In its initial development, single-cell Hi-C (scHi-C) adopted an approach employed by the nuclear ligation assay by performing cross-linking, restriction digestion, and ligation within intact nuclei, after which it isolated individual nuclei and proceeded through the rest of the Hi-C methodology (Cullen et al., 1993; Nagano et al., 2013). In situ bulk Hi-C subsequently drew upon similar inspiration by revising the original Hi-C protocol to perform ligation within permeabilized intact nuclei (Rao et al., 2014). These protocol adjustments enable ligation in smaller volumes, reduce the frequency of spurious contacts, and improve digestion efficiency, resulting in cleaner and higher-resolution data (Nagano, Várnai, et al., 2015; Rao et al., 2014). Subsequent scHi-C derivatives, such as single-nucleus Hi-C (snHi-C) and single-cell combinatorial indexed Hi-C (sciHi-C), have generated multiplexed libraries using tagmentation and indexing (Ramani et al., 2017), improved nuclear sorting efficiency using FACS (Nagano et al., 2017), or minimized contact loss using in situ whole-genome amplification (Flyamer et al., 2017). Given the population-averaged nature of genomic interactome data from 3C-based methods, scHi-C has proven instrumental in distilling cell-to-cell structural heterogeneity, identifying rare cellular subpopulations, and understanding how different levels of organization interact (Nagano et al., 2013; Stevens et al., 2017; Ulianov, Tachibana-Konwalski, & Razin, 2017). For example, Nagano et al. found that transcriptionally active domains hundreds of kb to megabases in size localize to the peripheries of territories hundreds of Mb in size, and Stevens et al. reported that while TADs and loops substantially vary from cell-to-cell, compartments, lamina-associated domains, and active enhancers and promoters do not. Notably, scHi-C has also disentangled cell-cycle dynamics governing features of 3D nuclear organization (Nagano et al., 2017) and revealed developmentally-linked chromatin reorganization in the oocyte-to-zygote transition, such as the presence of TADs and loops but not compartments in zygotic maternal chromatin (Flyamer et al., 2017). However, scHi-C is limited in its ability to detect any given contact because it only captures data from a cell’s one (zygotic studies) or two (somatic studies) alleles for any given locus and because the likelihood of detecting an interaction is low (Nagano, Lubling, et al., 2015; Tan, Xing, Chang, Li, & Xie, 2018). Thus, scHi-C faces challenges in separating technical noise from biological variation, and the sparsity of the contact matrix for any given cell presents a challenge in data analysis and interpretation (Lähnemann et al., 2020; Ulianov et al., 2017; Zhu & Wang, 2019).

#### ChIP-based methods

Interest in the protein-DNA interactions contributing to the 3D genome has spawned the inclusion of chromatin immunoprecipitation (ChIP) methodology into chromosome conformation capture protocols. First introduced in 2005, ChIP-loop (also known as ChIP-3C) modifies the 3C protocol by enriching ligated fragments for contact with a protein of interest (Horike, Cai, Miyano, Cheng, & Kohwi-Shigematsu, 2005). As a “one versus one” method (Figure 1b), ChIP-loop was initially used to investigate chromatin interactions bound by MeCP2, SATB1, or ERα (S. Cai, Lee, & Kohwi-Shigematsu, 2006; Carroll et al., 2005; Fullwood & Ruan, 2009; Horike et al., 2005). Substituting in cloning-based contact analysis in lieu of PCR amplification yields 6C (combined 3C-ChIP-cloning), a method first used to examine the role of EZH2 in mediating long-range contacts (Tiwari, Cope, McGarvey, Ohm, & Baylin, 2008). However, difficulty quantifying ChIP enrichment of inherently noisy 3C data made distinguishing specific interactions from nonspecific false positives in ChIP-loop-based techniques challenging (Fullwood & Ruan, 2009). The whole-genome (“all versus all,” Figure 1b) adaption of ChIP-loop methods, called chromatin interaction analysis by paired-end tag sequencing (ChIA-PET), increases the signal-to-noise ratio (SNR) by using sonication to fragment DNA, adds linker sequences between ligated fragments for ease of extraction, and utilizes adapters for high-throughput paired-end sequencing (Fullwood et al., 2009). Examples of early applications of ChIA-PET include investigations of the chromatin interactomes of ERα (Fullwood et al., 2009) and CTCF (Handoko et al., 2011).

By virtue of their enrichment of protein-centered chromatin interactions, genome-wide ChIP-based conformation capture methods are capable of recapitulating key features of the 3D genome (e.g., TADs and loops) and achieving finer resolution than Hi-C (e.g., by enriching for determinants of organization, such as CTCF and cohesin). Given similarities in methodology, subsequent advances in whole-genome ChIP-based techniques have often drawn from parallel advancements in Hi-C-based techniques. For example, HiChIP, which exhibits improved sensitivity to DNA contacts and lowered input material requirements relative to ChIA-PET, was built by leveraging principles of in situ Hi-C (Mumbach et al., 2016). More recently, multiplex chromatin-interaction analysis via droplet-based and barcode-linked sequencing (ChIA-Drop) has been developed by utilizing microfluidics to partition cross-linked chromatin complexes into gel-bead-in-emulsion (GEM) droplets for subsequent barcoding, amplification, and sequencing (Zheng et al., 2019). Able to probe chromatin interactions with greater precision and resolution than ChIA-PET, ChIP-Drop has been used to explore transcriptionally relevant promoter-centered interactions and shows promise for uncovering novel single-molecule-resolution multi-way contacts (Kempfer & Pombo, 2020; Zheng et al., 2019).

#### Capture-based methods

With 3C-based methods at the time unable to map *cis* interactions at a sufficiently high resolution to capture enhancer-promoter contacts in a high-throughput manner, Capture-C was developed in 2014 as a new approach to exploring *cis* regulation (Hughes et al., 2014). Combining 3C, oligonucleotide capture technology (OCT), and high-throughput sequencing, Capture-C utilizes RNA biotinylated oligo probes to enrich for DNA fragments containing viewpoints of interest. As such, Capture-C can be applied to probe a specific contiguous region (a “many versus many” experiment similar to 5C) or, if probes are designed for, for example, all promoters, Capture-C can serve as a massively parallel 4C (“many versus all”) experiment (Figure 1b). The initial application of Capture-C to the promoters of genes of interest demonstrated the method’s ability to elucidate general principles of *cis* regulation and link single-nucleotide polymorphisms (SNPs) in distal elements to expression changes in their cognate genes (Hughes et al., 2014). Soon after, similar target sequence enrichment was applied to Hi-C libraries to yield Capture Hi-C (CHi-C), further facilitating the discovery of novel long-range promoter contacts (Jäger et al., 2015; Mifsud et al., 2015). Both Capture-C and Capture Hi-C have been applied to investigate gene loci implicated in limb development, demonstrating the phenotypic implications of disrupting enhancer-promoter chromatin structure and uncovering two regimes of chromatin folding: one associated with CTCF/RAD21 binding that is stable across tissues, and another that correlates with tissue-specific changes in chromatin microarchitecture (Andrey et al., 2017; Paliou et al., 2019). Similarly, Capture Hi-C and 4C-seq testing of intra-TAD and inter-TAD insulation in response to genomic duplications revealed the formation of new chromatin domains with pathogenic consequences, further linking structure to phenotype (Franke et al., 2016).

Noting that Capture-C’s reliance upon oligonucleotides synthesized on microarrays yielded high costs per sample for small experimental designs and that the Capture-C method was not sensitive enough to detect weak interactions, researchers developed next-generation (NG) Capture-C as a solution (Davies et al., 2016). The NG Capture-C method performs capture using DNA (rather than RNA) biotinylated oligos, introduces a second round of capture, and pools multiple independent 3C libraries for processing in a singular reaction, thus improving sensitivity and throughput. The latest iterations of Capture-C design, Nuclear-Titrated (NuTi) Capture-C and Tiled-C, further advance the resolution and efficacy of Capture-C-based methods (Downes et al., 2020; Oudelaar et al., 2020). NuTi Capture-C isolates 3C libraries from intact nuclei by separating 3C libraries into nuclear and non-nuclear fractions post-ligation and utilizes shorter oligonucleotide probes (shortened from 120 to 50 bp). Tiled-C uses a panel of capture oligonucleotides tiled across all restriction fragments of a region of interest; combined with an optimized protocol which minimizes losses and maximizes library complexity, this allows the method to generate high-resolution contact maps from inputs of very few cells (as low as 2,000). In its initial application, Tiled-C followed the nuclear architecture of mouse erythroid genes of interest through in vivo erythroid differentiation and revealed that structural reorganization within TADs and the emergence of E–P contacts occurs during differentiation, suggesting that chromatin architecture and gene activation are linked (Oudelaar et al., 2020).

Traditional 3C-based methods are limited to detecting pairwise contacts, preventing them from determining the interdependence of multi-way chromatin contacts commonly found in biological systems (Kempfer & Pombo, 2020). For instance, if loci A and B both interact with locus C, do they do so in a mutually exclusive, mutually dependent, or independent manner? 3C-based methods such as chromosomal walks (Olivares-Chauvet et al., 2016) and the concatemer ligation assay (Darrow et al., 2016) began addressing this question by 2016 and, looking to probe multi-way contacts with single-allele resolution, researchers created Tri-C in 2018 (Oudelaar et al., 2018). Employing sonication of ligated fragments to ~450 bp in size, Tri-C creates libraries where a majority of fragments contain multiple ligation junctions that can then be captured by OCT, PCR amplified, and sequenced. Examination of domains containing mouse globin loci using Tri-C reveals regulatory hubs containing multiple enhancers and promoters, as well as heterogeneous patterns of CTCF interaction indicating highly variable chromatin domain formation (Oudelaar et al., 2018). Similarly, multi-contact 4C (MC-4C), published concurrently with Tri-C, has been used to analyze three-way contacts within a region of interest and disentangle cooperative interactions from competitive or random interactions (Allahyar et al., 2018). Recently, the combination of ATAC-seq (Assay for Transposase-Accessible Chromatin using sequencing), i4C-seq, CRISPR/Cas9 modification, and single-molecule RNA FISH to study mechanisms of action underlying cytokine-activated enhancer activity has yielded a cross-linking-free variant of MC-4C and allowed multi-way interactions spanning TAD boundaries to be studied (Weiterer et al., 2020).

### 2.3 Non 3C-based technologies to map the 3D genome

#### SPRITE and GAM

While the exploration of the 3D genome has largely been propelled by 3C-derived technologies, other 3D mapping methods have revealed unique insights about genome organization by capturing interactions not preserved by 3C-based methodologies. For example, despite being the gold standard in chromosome conformation capture, Hi-C is limited in its reliance on restriction digestion and proximity ligation; restriction digestion imposes a limit on the resolution of captured data that can be achieved, while proximity ligation biases analysis to primarily pair-wise interactions, may be inefficient, and can invite the inclusion of spurious contacts. Developed as a method for investigating higher-order organizational interactions not captured by proximity ligation, split-pool recognition of interactions by tag extension (SPRITE) addresses some of Hi-C’s shortcomings and reveals hubs of interaction within the nucleus (Quinodoz et al., 2018). In a nutshell, the SPRITE method cross-links DNA, RNA, and proteins within a cell, isolates the nucleus, fragments chromatin, sequentially barcodes interacting molecules within each individual complex through several rounds of split-pooling, and exhaustively sequences the barcoded molecules to reconstruct the interactome. By focusing on hubs of cross-linked interactions and forgoing both restriction digestion and proximity ligation, SPRITE is able to probe multi-way contacts across a wide range of nuclear distances at high resolution. Free from a resolution ceiling imposed by RE digestion, SPRITE performs chromatin fragmentation using sonication and DNase digestion; in its initial application, this digestion was optimized to produce DNA fragments 150–1,000 bp in length (Quinodoz et al., 2018). With ~1.5 billion sequencing reads, SPRITE achieved similar kilobase-scale resolution as high-resolution Hi-C and recapitulated known structures of nuclear architecture. Using SPRITE, Quinodoz et al. also observed long-range nuclear interactions in which gene-dense and highly transcribed regions preferentially localize around nuclear speckles while gene-poor and transcriptionally inactive regions localize around the nucleolus. In addition to its ability to detect higher-order genomic organization beyond pairwise interactions, SPRITE can simultaneously explore both the DNA and RNA interactomes, requires fewer input cells than Hi-C, and captures long-range interactions involving actively transcribed enhancers and promoters rarely seen in Hi-C data; conversely, however, it is also a more laborious, lower-throughput process than Hi-C and the efficiency of serially ligating small oligonucleotides for barcoding remains uncharacterized (Fiorillo et al., 2020; Kempfer & Pombo, 2020).

Another technique, Genome Architecture Mapping (GAM), overcomes the limited ability of 3C-based methods to capture multi-way simultaneous interactions (e.g., triplet contacts) and other aspects of organization such as compaction and association with the nuclear periphery (Beagrie et al., 2017). GAM measures chromatin contacts by thinly cryo-sectioning fixed cells, isolating nuclear profiles, and extracting, amplifying, and sequencing the DNA within many such randomly sliced profiles to map the co-segregation rates of all possible pairs of genomic loci using the SLICE (statistical inference of co-segregation) analysis method. The number of collected nuclear slices determines the resolution of GAM data sets, with 400 nuclear slices sequenced with ~1 million reads per slice yielding comparable pairwise contact resolution (30 kb) to Hi-C (Kempfer & Pombo, 2020). As no maximum resolution limit has been achieved for GAM, larger datasets should yield finer resolution data in the future. Successfully benchmarked against Hi-C maps, GAM has shown that enhancers and active genes are enriched among specifically interacting genomic regions, with particularly strong enrichment at transcriptional start and end sites (TSSs & TESs; Beagrie et al., 2017). When compared to Hi-C, GAM is advantageous for its lack of RE digestion, proximity ligation, or chromatin fragmentation and exhibits a superior robustness to noise at large genomic distances, requires fewer input cells, and is well-suited for analysis of complex tissues (e.g., patient biopsies; Beagrie et al., 2017; Fiorillo et al., 2020). Moreover, like SPRITE, GAM’s detection of long-range contacts of transcriptionally active enhancers and promoters outpaces Hi-C, as such contacts are difficult to discern in Hi-C data. However, GAM is limited by its dependence upon specialized equipment and training (e.g., for fine cryo-sectioning) and increased complexity of data interpretation (Kempfer & Pombo, 2020).

Though not a measure of chromosome conformation (and accordingly not discussed here in depth), ionizing radiation-induced spatially correlated cleavage of DNA with sequencing (RICC-seq) is another noteworthy technique that has revealed nucleosome-level folding and local structure (Risca, Denny, Straight, & Greenleaf, 2017). Alternatively, the development of i3C, accompanied by the TALE-iD method for validating i3C and i4C interactions via TAL-effector-directed methylation of target enhancers, provided an early framework for cross-linking-free chromosome conformation capture (Brant et al., 2016). This approach was recently further developed in the combination of DNA adenine methyltransferase identification (DamID) with chromosome conformation capture in the DamC method, making it possible to study 3D genome architecture without cross-linking and proximity ligation (Redolfi et al., 2019).

#### RNA-based methods

Though long the primary focus in nuclear architecture, the DNA interactome is one structural piece among many in the nucleus. In recent years, advances in techniques probing DNA–DNA and DNA-protein contacts have been adapted to study the DNA–RNA and RNA–RNA interactomes. For the sake of brevity, we touch upon only a few “all versus all” technologies in this area. Methods for studying DNA–RNA interactions, such as chromatin-associated RNA sequencing (ChAR-seq) (J. C. Bell et al., 2018), global RNA interactions with DNA by deep sequencing (GRID-seq) (Li et al., 2017), and mapping RNA-genome interactions (MARGI) (Sridhar et al., 2017; Wu et al., 2019) follow the same core workflow: cross-link cells, ligate a linker to cross-linked RNA, reverse transcribe, proximity ligate the cDNA-linker to DNA, and isolate ligated pairs for amplification and paired-end sequencing. Differences between these techniques arise in the design of the linker or “bridge” and the ordering of steps, but all have been instrumental in identifying novel transcript-DNA interactions and distilling the contacts structurally underpinning transcription. RNA–RNA methods, such as RNA hybrid and individual-nucleotide resolution ultraviolet cross-linking and immunoprecipitation (hiCLIP) (Sugimoto et al., 2015) and RNA in situ conformation sequencing (RIC-seq) (Z. Cai et al., 2020), fix and cross-link cells and often utilize proximity ligation to link RNA–RNA-contacts mediated by RNA-binding proteins (RBPs). With RIC-seq achieving single-nucleotide resolution and revealing the necessary role of ncRNA in looping at the *MYC* and *PVT1* loci (Z. Cai et al., 2020), we anticipate the rapid discovery of more RNA-dependent nuclear organization in coming years.

#### Microscopy-based methods

Beyond the sequencing-based methods discussed above, microscopy-based methods have been instrumental in visualizing nuclear architecture, measuring characteristics of folding (e.g., compaction), identifying chromatin domains, and distilling cellular heterogeneity. With advancements in super-resolution microscopy and synthetic oligonucleotide design, techniques such as fluorescence in situ hybridization (FISH), OligoPAINT, three-dimensional assay for transposase-accessible chromatin-photoactivated localization microscopy (3D ATAC-PALM), stochastic optical reconstruction microscopy (STORM), optical reconstruction of chromatin architecture (ORCA), and live-cell imaging have uncovered structural insights and multiway interactions not captured by the other methods discussed here (Alexander et al., 2019; Beliveau et al., 2012; Chen et al., 2018; Marbouty et al., 2015; Mateo et al., 2019; Rust, Bates, & Zhuang, 2006; Xie et al., 2020). For the sake of brevity, we do not discuss these microscopy-based methods in detail here and instead refer the reader to excellent reviews on the topic (Boettiger & Murphy, 2020; Cattoni, Valeri, Le Gall, & Nollmann, 2015; Kempfer & Pombo, 2020; Lakadamyali & Cosma, 2020).

## 3 MECHANISTIC UNDERPINNINGS OF NUCLEAR ARCHITECTURE

Though the development of 3C-based technologies uncovered features of chromatin organization, understanding the biological mechanisms underlying the features necessitated further experimentation. The past half-decade in particular has seen a renaissance in our mechanistic understanding of 3D genome organizational features as researchers have unearthed the roles of key proteins, forces, and mechanisms and developed advanced models explaining DNA conformation. Specifically, landmark discoveries that have shaped the field’s current biological grounding involve the elucidation of the roles of CTCF and cohesin, the loop extrusion model, compartmentalization, and the interplay of different forces across levels of organization.

### 3.1 The roles of CTCF and cohesin

Although the development of Hi-C established TADs as hallmarks of genome organization, uncertainty remained over how TADs are formed, maintained, and regulated. Interest in mechanisms capable of locally confining genomic regions from one another began drawing upon decades of research into transcriptional regulation. Of particular interest were insulators, regulatory elements first recognized in the early 1990s for their ability to act as local barriers to *cis*-acting elements (e.g., blocking the action of distal enhancers on promoters) (West, Gaszner, & Felsenfeld, 2002). By the turn of the century, CTCF had become a poster child as the first protein recognized as binding enhancer-blocking insulators in vertebrates (A. C. Bell, West, & Felsenfeld, 1999, 2001; Ohlsson, Renkawitz, & Lobanenkov, 2001; West et al., 2002). However, its mechanistic role in insulator activity remained a mystery (Ohlsson et al., 2001), making it a promising candidate for a possible role in TAD creation and modulation.

The first TAD papers in 2012 examined a host of factors as possible determinants of TAD formation, among which were CTCF binding sites found to be enriched at TAD boundaries (Dixon et al., 2012; Nora et al., 2012). However, noting that CTCF has many nonboundary bindings sites, the papers rationalized that the CTCF and cohesin proteins could not be primary determinants of TAD formation. Attempting to ascertain the importance of CTCF and cohesin loss on TADs, researchers performed partial depletion experiments in 2013 and 2014. CTCF depletion reduced insulation between adjacent TADs (Zuin et al., 2014) while cohesin depletion diminished long-range genomic interaction (Seitan et al., 2013; Zuin et al., 2014), relaxed chromatin domains (Sofueva et al., 2013), and dose-dependently affected cellular phenotype (Viny et al., 2015). Collectively, these results suggested that while CTCF and cohesin play important roles in nuclear architecture, they are not fundamentally necessary for TAD formation. However, subsequent ultra-high-resolution Hi-C analysis of the mammalian genome found that >86% of loop boundaries are enriched for CTCF and cohesin binding, implying a central role for both CTCF and cohesin in TAD formation (Rao et al., 2014). Critically, ChIP-seq, CRISPR genome editing, and Hi-C analyses established that the polarity of CTCF sites is a determinant of loop formation (de Wit et al., 2015; Guo et al., 2015; Rao et al., 2014; Sanborn et al., 2015; Tang et al., 2015; Vietri Rudan et al., 2015). CTCF’s consensus DNA motif is not a palindrome; instead, it has polarity or directionality that is then also imparted to the CTCF protein. When considering a pair of CTCF sites, there are four possible conformations for their relative polarities: convergent (both sites oriented inward), divergent (both sites oriented outward), and tandem (both sites facing the same direction along either strand of DNA). Assuming looping to be unaffected by CTCF polarity, we would expect ~25% of CTCF loop anchors to be convergently oriented; however, analysis of CTCF anchor motifs reveals that 65%–92% (depending on the bio-informatic analysis) of CTCF loops anchors are convergent (de Wit et al., 2015; Guo et al., 2015; Rao et al., 2014; Tang et al., 2015), showing strong enrichment that allows site polarity to be a robust predictor of looping interactions (Sanborn et al., 2015). This preference for CTCF binding site polarity is known as the convergent rule and violating the rule (e.g., via CRISPR inversion of CTCF sites), though not a guaranteed predictor, disrupts chromosomal topology (de Wit et al., 2015; Guo et al., 2015; Sanborn et al., 2015).

It was not until 2017 that the roles of CTCF and cohesin in TAD formation and maintenance were clarified. Nearcomplete auxin-inducible degron (AID) degradation of CTCF established that CTCF loss reduces TAD insulation and that CTCF acts in a dose-dependent manner, though with some boundaries only being moderately affected (Nora et al., 2017; Wutz et al., 2017). Ramifications of CTCF loss on looping, such as a partial loss of TADs, primarily manifest following >80% CTCF degradation and accordingly were not captured by the previously discussed partial depletion experiments. Cohesin depletion via AID-mediated degradation of cohesin (Rao et al., 2017; Wutz et al., 2017) or deletion of NIPBL (Schwarzer et al., 2017) effectively eliminates all TADs, a stronger effect than observed for CTCF loss and one which points to the essentiality of cohesin in loop domain formation. CRISPR knockout (Haarhuis et al., 2017) of cohesin DNA release factor WAPL or RNAi depletion (Wutz et al., 2017) of WAPL and the PDS5A & PDS5B proteins causes violation of the convergent rule and reveals the factors’ roles in modulating cohesin unloading from DNA as accessory proteins. Specifically, depletion of either WAPL or the PDS5 proteins increases cohesin processivity and DNA residence time, resulting in fewer but larger TADs and chromatin compaction visible as highly compacted “vermicelli” (Tedeschi et al., 2013). Furthermore, snHi-C analysis of mice knockout embryos for cohesin functionality affirmed the vital structural roles played by cohesin and WAPL (Gassler et al., 2017). Collectively, these advancements shifted the 3D genome field’s focus toward CTCF, cohesin, and their associated proteins as determinants of long-range genome looping.

### 3.2 The loop extrusion model

The discovery of the critical roles CTCF and cohesin play in TAD formation motivated investigations into how proteins could mechanistically facilitate the formation of megabase-scale DNA loops. Speculation that some form of “DNA reeling” may explain loop folding dates back as far as 1990 (Riggs, Holliday, Monk, & Pugh, 1990), and modeling work conducted in the wake of the first observation of TADs theorized that a hypothetical DNA-loop-extruding enzyme could create and anchor loop domains (Alipour & Marko, 2012). The modeled enzymes were based off of condensins, SMC complexes similar to cohesin that reorganize mitotic chromosomes (Yatskevich, Rhodes, & Nasmyth, 2019). The emergence of CTCF and cohesin as predictors of loop formation and the CTCF convergent rule ultimately led to the loop extrusion (LE) model (Nichols & Corces, 2015). First proposed in 2015, the LE model was born out of mathematical simulations and polymer modeling suggesting that loops are formed as a *cis*-acting LE factor (e.g., an SMC complex) “extrudes” DNA until it stalls at a boundary element (e.g., CTCF proteins) (Fudenberg et al., 2016; Sanborn et al., 2015). Specifically, the LE model proposes that an LE factor (such as cohesin) lands on DNA and extrudes a loop uni- or bi-directionally until it encounters an occupied CTCF binding site, where a loop is then stabilized. Since CTCF and cohesin turnover on DNA is dynamic, it is likely that such extruded DNA loops would be similarly dynamic (Hansen, Pustova, Cattoglio, Tjian, & Darzacq, 2017). Polymer simulations of the chromatin fiber under the assumptions of this model successfully recreated TADs and other known features of nuclear architecture, including nested TADs, stripes/flames (lines of increased contact frequency at the sides of a TAD), preferential intra-TAD contact, and TAD merging upon boundary deletion (Fudenberg, Abdennur, Imakaev, Goloborodko, & Mirny, 2017; Fudenberg et al., 2016). An elegantly simple model, loop extrusion was postulated without any direct biochemical evidence to support cohesin’s ability to extrude DNA nor CTCF’s ability to block cohesin extrusion in an orientation-specific manner; accordingly, the model initially received significant skepticism. Nevertheless, evidence quickly began to emerge from imaging studies (below) and, indirectly, from Hi-C studies of condensin in bacteria (Tran, Laub, & Le, 2017; X. Wang, Brandão, Le, Laub, & Rudner, 2017).

The first test of loop extrusion came from in vitro single-molecule imaging studies. Initial examinations of single-molecule cohesin dynamics reported passive diffusion of cohesin along naked DNA and that translocation occurs in a manner suggesting topological entrapment, with both transcriptional machinery and CTCF constraining movement (Davidson et al., 2016; Kanke, Tahara, Huis in’t Veld, & Nishiyama, 2016; Stigler, Qamdere, Koshland, & Greene, 2016); however, these papers failed to detect active loop extrusion by the cohesin complex. Subsequent characterization of the molecular motor activity of condensin with a DNA curtain assay suggested that cohesin may similarly be capable of rapid and processive ATP-dependent directional translocation (Terakawa et al., 2017). Direct real-time visualization of condensin-driven extrusion of loops containing tens of kilobases of DNA soon after provided compelling evidence for the LE model (Ganji et al., 2018). Revisiting cohesin, researchers have recently observed cohesin-driven DNA loop extrusion in vitro for the first time (Davidson et al., 2019; Golfier, Quail, Kimura, & Brugues, 2020; Y. Kim, Shi, Zhang, Finkelstein, & Yu, 2020). DNA curtain assays have uncovered that loop extrusion by cohesin is dependent upon not only ATP-hydrolysis but also upon association with NIPBL, a HAWK (HEAT repeat protein associated with kleisins) now known to be an essential part of the extruding holoenzyme (Davidson et al., 2019; Y. Kim et al., 2020; Shi, Gao, Bai, & Yu, 2020). This dependence upon NIPBL explains the absence of extrusion in the earlier in vitro single-molecule cohesin experiments since NIPBL was not included in experimental conditions. Loop extrusion has also been demonstrated in *Xenopus* egg extracts, confirming a role for cohesin and condensin activity in interphase and mitosis, respectively (Golfier et al., 2020). Thus, in the half-decade since its proposition, the loop extrusion model has aligned with novel biochemical insights about the 3D genome and emerged as the leading model for the mechanistic underpinnings of TADs and other loop domains.

Despite these recent breakthroughs, loop extrusion is far from being completely understood. Though we now know that cohesin can extrude loops of naked DNA in vitro and that CTCF likely acts as a boundary factor, the mechanism by which CTCF stalls cohesin in a polarity-dependent manner is unknown and a topic of intense current study (Hansen, 2020). Uncertainty also remains as to whether cohesin extrusion occurs as a monomer (Davidson et al., 2019) or a dimer (Y. Kim et al., 2020), as well as the precise nature of the topological interaction between DNA and the extrusion complex. Finally, our current understanding of loop extrusion is grounded entirely in in vitro experimentation, with extrusion yet to be demonstrated in vivo and on chromatin. We anticipate these looming questions to be resolved in coming years as the field tackles this mechanistic frontier of chromatin organization and refines the loop extrusion model.

### 3.3 Levels and mechanisms of genomic organization

Equipped with 3C-based technologies capable of analyzing genomic organization at various resolutions and beginning to understand what loop extrusion can (and cannot) explain, researchers have started unraveling the complexity of the 3D genome across levels of organization and the competing forces at play. At the scale of whole chromosomes (hundreds of megabases), chromosomal territories have been known to segregate chromosomes into preferred locales within the nucleus for over a century (Cremer & Cremer, 2010). Beginning with genomic compartments, which separate chromatin into two preferentially self-interacting, megabase-scale groups characterized by transcriptionally active euchromatin (A) or inactive heterochromatin (B), the field has rapidly uncovered features of finer and finer scales of chromatin organization. Manifesting on the scale of hundreds of kilobases to a few megabases, TADs demarcate regions in which intra-TAD DNA–DNA contact frequencies tend to be at least two-fold higher than inter-TAD contact frequencies. Within these TADs may lie nested TADs or “subTADs,” which themselves separate intra-TAD regions into regions on the order of tens to hundreds of kilobases of enhanced self-interaction (Beagan & Phillips-Cremins, 2020; Phillips-Cremins et al., 2013). TAD and subTAD boundaries may be characterized by stripes (also called flames, flares, or tracks), which likely result from the extrusion activity forming the domain (Fudenberg et al., 2017). Similar to TADs and subTADs, compartments can also have nested subcompartments contributing to the intricacies of organization (Rao et al., 2014; Rosencrance et al., 2020; Rowley et al., 2019; Sati et al., 2020); for instance, a large A compartment can have a smaller B compartment within it. Finally, enhancer-promoter and promoter-promoter dots and stripes (also called E–P and P–P loops/interactions), generally appearing at the 5–500 kb scale, link active enhancers and promoters and thereby impart specific functional consequence to looping activity. Importantly, these scales of organization all simultaneously contribute to the complexity of nuclear architecture.

Intriguingly, however, it has become apparent that the forces contributing to the formation of these observed organizational features are not entirely mutually exclusive (Box 1). In particular, studies have explored the interplay between chromatin looping and compartmentalization. CTCF depletion reduces TAD insulation and disrupts a subset of TADs but has no substantive effect on compartments, indicating that compartmentalization is driven by other factors (Nora et al., 2017; Wutz et al., 2017). Cohesin removal via AID degradation (Rao et al., 2017; Wutz et al., 2017), deletion of RAD21 (Gassler et al., 2017), or deletion of NIPBL (Schwarzer et al., 2017) shows that compartmentalization becomes more prominent even as TADs and loop domains are lost. By increasing cohesin processivity, WAPL depletion not only increases TAD size but also makes compartments less prominent (Gassler et al., 2017; Haarhuis et al., 2017; Wutz et al., 2017). Collectively, these results suggest that two major forces, namely loop extrusion and compartmentalization, are at play in genomic organization and that the former antagonizes the latter. While the biophysics of loop formation have been attributed to the LE model, the biophysical mechanisms that govern compartmentalization remain very poorly understood. One explanation for the observed one-way antagonization is that loop extrusion is an active force reshaping the chromatin landscape whereas compartmentalization is a more passive force; if so, active LE could maintain the separation of chromosomal segments that may otherwise slowly segregate according to their compartmental identities. A leading explanation for such passive compartmentalization is phase separation, the self-organization of a heterogeneous mixture into distinct phases with different constituent compositions (Erdel & Rippe, 2018; Nuebler, Fudenberg, Imakaev, Abdennur, & Mirny, 2018). In cells, microphase separation is believed to contribute to the formation of “chromatin bodies” by polymer-polymer phase separation (PPPS) of proteins bridging proximally located nucleosomes or by liquid–liquid phase separation (LLPS) of soluble molecules multivalently interacting with chromatin (Erdel & Rippe, 2018). Lending credence to microphase separation’s role in passive compartmentalization, researchers have observed an inherent tendency of segments of A/B chromatin to act and separate like a block co-polymer (Hildebrand & Dekker, 2020). Moreover, polymer simulations assuming principles of microphase separation of block copolymers have successfully recapitulated Hi-C data and depletion experiments capturing both LE and compartmentalization (Nuebler et al., 2018).

Elucidating the mechanisms and forces at play in genomic organization is key to building a better understanding of how the features we observe in chromosome contact maps relate to function. Although TADs spatially define genomic regions according to self-interaction, it is crucial to remember that they represent population-averaged contact frequencies that rarely have a corollary domain within any individual cell (Bintu et al., 2018; Finn et al., 2019; Finn & Misteli, 2019). By contrast, compartmental interactions are present in most single cells and can be correlated with the general transcriptional activity of large swathes of DNA (S. Wang et al., 2016); however, their lack of specificity makes drawing strong functional consequence for any given gene difficult. It is the finer levels of organization (e.g., E–P loops), then, that are more directly implicated in modulating gene expression and function. Though Hi-C is the gold standard in genome-wide conformation capture, a genome-wide genetic screen mapping 470 functional enhancer-gene pairs using an expression quantitative trait locus (eQTL) framework showed that the majority of enhancer-promoter pairs are not identified as contacts by Hi-C (Gasperini et al., 2019). Thus, technologies capable of reliably visualizing and disentangling the biological mechanisms at play in local genome organization are critically important for the functional advancement of the field. One such technology, Micro-C, maps genomes at unprecedented resolution and merits further discussion.

## 4 MICRO-C: A FRONTIER IN FINE GENOME MAPPING

Despite Hi-C’s dominance as the flagship high-throughput chromosome conformation capture technology, researchers began reaching the limits of its ability to capture fine-resolution (~1 kb) genome architecture by the mid-2010s (Rao et al., 2014). Local DNA loops and dots were only visible following billions of Hi-C sequencing reads, an expensive threshold for substantial exploration of small-scale chromatin architecture. The intrinsic limitations to resolution imposed by REs spurred the development of alternative methods for probing fine genome folding features such as DNase Hi-C, a Hi-C derivative employing DNase I chromatin digestion over restriction digestion to improve capture resolution to 1–10 kb (Ma et al., 2015, 2018). Another such development was Micro-C, a Hi-C derivative with nucleosome-level (~200 bp) resolution, in 2015 (Hsieh et al., 2015). First developed in yeast *(Saccharomyces cerevisiae)* (Hsieh et al., 2015; Hsieh, Fudenberg, Goloborodko, & Rando, 2016) and most recently optimized for mammalian chromosome conformation capture (Hansen et al., 2019; Hsieh et al., 2020; Krietenstein et al., 2020), Micro-C has uncovered novel features of chromatin architecture and facilitated an analysis of the biological mechanisms underpinning DNA folding.

**FIGURE 3:**
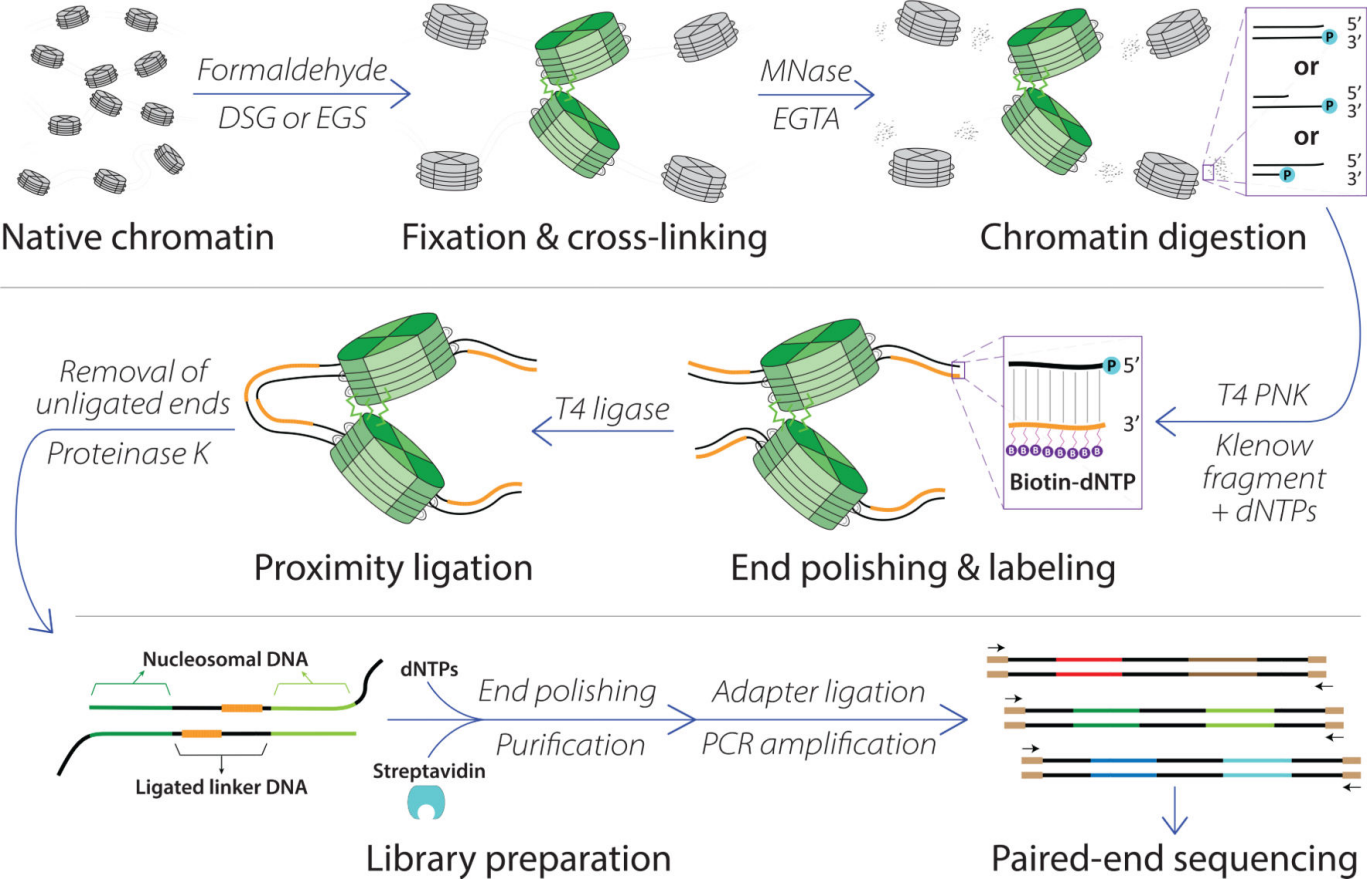
The Micro-C protocol. Major steps in the Micro-C method are shown. Cells are first chemically fixed using formaldehyde and cross-linked using a protein–protein cross-linker (shown as bright green jagged lines) such as disuccinimidyl glutarate (DSG) or ethylene glycol bis(succinimidyl succinate) (EGS). MNase digestion cleaves DNA into mononucleosomes and is inactivated by EGTA. The resulting DNA ends are blunted, polished, and labeled with biotin. DNA is proximity ligated, cross-links are reversed, and nonligated products are removed through streptavidin purification. The Micro-C library is prepared for paired-end sequencing by sequencing adapter ligation and PCR amplification. Data processing (not shown) can be performed with a data analysis pipeline similar to Hi-C

Micro-C begins by fixing and chemically cross-linking cells using formaldehyde (protein-DNA and protein-protein interactions) and disuccinimidyl glutarate (protein-protein interactions); this double cross-linking is unique to the Micro-C method (Figure 3). Cell membranes are subsequently solubilized, and the cross-linked chromatin is digested with micrococcal nuclease (MNase) down to nucleosome-level resolution; the choice of MNase digestion over restriction digestion distinguishes Micro-C from its sister methods, and the relevance of this alteration will be discussed shortly. Post-digestion sticky ends are then blunted with biotinylated dNTPs, and proximity ligation links the DNA of proximally located nucleosomes. Following reverse cross-linking and DNA purification, gel electrophoresis and extraction allows the size-selection of ligated di-nucleosomal DNA. Finally, adapter ligation and PCR amplification generate the Micro-C library, and sequencing quantifies the genome-wide DNA interactome. Sequencing data is processed the same way as Hi-C data, with read mapping, fragment assignment, filtering, binning, and bias correction steps; for further guidance on chromosome conformation capture data analysis pipelines, we refer the reader to a comprehensive review (Lajoie, Dekker, & Kaplan, 2015).

### 4.1 Micro-C captures finer organizational resolution than other methods

The hallmark innovation of Micro-C is the replacement of RE digestion with micrococcal nuclease (MNase) digestion. This protocol alteration, though seemingly minor, shifts the resolution of conformation capture to finer scales. REs are endonucleases that cleave DNA at their recognition sequences, which are usually 4 bp, 6 bp, or 8 bp in length. When performing genome-wide analyses (e.g., Hi-C), restriction digestion is therefore predicted to cleave the genome on average every 4^4^ = 256 bp or 4^6^ = 4,096 bp for 4-bp or 6-bp cutters, respectively; however, such an assumption is an oversimplification of the stochasticity of digestion. RE sites are not equally distributed throughout the genome, nor is all DNA readily accessible. The primary level of genomic organization is characterized by nucleosomes, in which 147 bp segments of DNA are tightly wound around histone proteins (Luger, Mäder, Richmond, Sargent, & Richmond, 1997); by contrast, more accessible “linker DNA” between two nucleosomes only spans ~20–90 bp in length, meaning that a majority of genomic DNA is affected by nucleosomal accessibility (Szerlong & Hansen, 2010). Chemical cross-linking of histones to nucleosomal DNA limits access to the DNA, thus strongly impeding RE digestion and precluding REs from cleaving all of their cognate genomic sequences (Chaya & Zaret, 2003). Consequently, there is widespread variation in fragment length between DNA cut sites in a fixed genome, causing features of chromatin architecture manifesting on the same or finer scales of resolution as the RE fragments to be partially or completely lost. For instance, Hi-C analysis performed with a 4 bp RE should yield near-nucleosomal-level resolution since it theoretically would cleave an average of every 256 bp. However, with the inaccessibility of RE sites affected by nucleosome cross-linking compounded by the nonrandom distribution of recognition sequences, the mean spacing between digested fragments substantially grows and the effective resolution of Hi-C data is degraded.

MNase, on the other hand, displays only mild DNA sequence preference (Allan, Fraser, Owen-Hughes, & Keszenman-Pereyra, 2012). An endo-exonuclease, MNase cleaves both ssDNA and dsDNA. With its enzymatic activity strictly Ca^2+^ dependent, MNase can be inactivated by calcium chelators for greater control over the extent of digestion (e.g., EGTA). Micro-C co-opts MNase’s indiscriminate digestion to achieve nucleosome-level resolution by taking advantage of local DNA accessibility. With DNA wound around nucleosomal histones largely protected from enzymatic degradation, MNase digestion begins at linker DNA (Voong, Xi, Wang, & Wang, 2017). By strictly regulating digestion time before EGTA-quenching the reaction, Micro-C is then able to maximize the retention of intact nucleosomes. Given the heterogeneity inherent in cell populations, determining an appropriate concentration for MNase digestion necessitates performing an MNase titration and visualizing the extent of nucleosomal digestion on an agarose gel. The optimal MNase concentration is one that produces 80% mononucleosomal/20% dinucleosomal DNA in the digestion window, a ratio tested to yield the best SNR and reproducibility of data (Hsieh et al., 2015). Despite the advantages conferred by its tunability, MNase also presents some shortcomings. The need to precisely control MNase digestion level makes application on heterogeneous populations (e.g., from patient samples) difficult. It also remains to be seen whether chromatin digestion to the 80% mononucleosomal/20% dinucleosomal level biases Micro-C maps toward well-positioned and easily accessible nucleosomes. Furthermore, minimizing undigested or un-ligated DNA contaminating the Micro-C library requires several purification steps, while gel-based separation and extraction of mononucleosomal DNA introduces a potential loss of information. We anticipate that future advancements in digestion control and purification will curtail these limitations of MNase use.

The resolution shift from restriction digestion to MNase digestion correspondingly shifts the effective resolution in Micro-C to finer scales than Hi-C. Micro-C captures TAD boundaries and compartments evident in Hi-C, whereas kilobase-scale features (e.g., stripes, E–P & P–P loops) are more clearly discernable in Micro-C maps compared to Hi-C maps (Figure 2). Conversely, however, Hi-C captures more long-range interactions beyond the megabase scale. This difference is derivative of the length of fragments generated by each method, as Hi-C generates longer fragments with a greater spread in size than Micro-C (Hsieh et al., 2016); these longer fragments are less capable of capturing fine-scale architecture than shorter fragments and thus give Hi-C poorer resolution than Micro-C (Hsieh et al., 2020). Contact frequency curves for Micro-C and Hi-C reflect these relative differences in captured information when mapped against genomic distance. When compared to Hi-C, a greater proportion of Micro-C reads fall at distances under 20 kb while, by the same token, a smaller proportion of Micro-C reads fall at distances above 10 Mb (Hsieh et al., 2020). This shift in captured contact frequency, visually apparent in Figure 2, pays dividends for the depth of sequencing necessary to visualize fine-scale genomic architecture using Micro-C. Detecting CTCF-anchored loops in Hi-C typically requires over 800 M unique sequencing reads; by contrast, Micro-C is able to detect loop structures with 80 M reads or less (Hsieh et al., 2020). This allows Micro-C to not only resolve loops identified in Hi-C with a fraction of the reads, but to also identify additional loops missed in Hi-C. For example, the initial application of Micro-C in mammalian cells analyzed 668 M reads and found 14,372 loops (Hansen et al., 2019), whereas high-resolution Hi-C analyzing 4.9B contacts found 9,448 loops using the same loop-calling algorithm (Rao et al., 2014). Recent application of high-resolution Micro-C reveals nearly fivefold more loops than Hi-C, as analysis of 2.64B Micro-C reads identified 29,548 loops (Hsieh et al., 2020) while analysis of 3.3B Hi-C reads identified only 6,006 loops in mESCs (Bonev et al., 2017). Thus, Micro-C is naturally inclined to probe finer genomic architecture than Hi-C and is poised to become a staple tool in the investigation of features below the level of TADs.

### 4.2 Micro-C uncovers novel biological insights about fine-scale architecture

Beyond defining novel features of the 3D genome, Micro-C has revealed valuable insights about the biological mechanisms underlying organization. The first application of Micro-C in mammalian cells examined the dependence of CTCF’s role in chromatin looping on an internal RNA-binding region (RBR_i_) and analyzed unique contacts in WT-CTCF and ΔRBR_i_-CTCF mESCs at medium resolution (668 M-694 M contacts) (Hansen et al., 2019). This early application of Micro-C to mESCs revealed two distinct classes of CTCF loops, namely those that are RBR_i_-dependent and those that are RBR_i_-independent. Two recent papers applying Micro-C at significantly higher resolution in mESCs, hESCs, and human fibroblasts describe mammalian chromatin structure in unprecedented detail; dissecting their major findings provides insight into the frontier of Micro-C technology (Hsieh et al., 2020; Krietenstein et al., 2020). In examining organizational features below the 20 kb scale across the genome, Hsieh et al. identify 29,548 loops and 136,223 novel fine-scale boundaries corresponding to smaller E–P & P–P stripes and domains than identified in Hi-C. Interestingly, ChIP-seq analysis indicates that the newly discovered fine-scale boundaries are predominantly (~85%) CTCF- and cohesin-negative, implying that they may be mediated by other CTCF-independent boundary factors and mechanisms modulating enhancer or promoter spatial dynamics (Di Giammartino et al., 2019; Mumbach et al., 2017; Zheng et al., 2019).

Characterizing transcription factors, architectural proteins, and regulators, Hsieh et al. (2020) also decouple the influence of dozens of key proteins on the strength and location of the identified fine-scale boundaries. Although CTCF and cohesin are strong predictors of boundary location, they are only moderate predictors of boundary strength; in fact, active promoters and *cis*-regulatory elements are the best predictors of boundary strength. Clustering analysis of boundaries reveals five overlapping subgroups distinguished by biochemical and functional features: (1) transcription-dependent, enriched for Pol II and TFs; (2) ES-cell-specific with Nanog and H3K4me1 enrichment; (3) YY1-related and found across cell types, with H3K27ac and Mediator enrichment; (4) repressive, enriched for bivalent chromatin related to the Polycomb complex; and (5) CTCF- and cohesin-mediated. These analyses highlight an abundance of boundary factors beyond CTCF, which largely remain to be characterized. Krietenstein et al., clustering dots visible in Micro-C but not Hi-C analysis, report similar classification of loops anchors into five groupings. Furthermore, examination of peak enrichment at insulators in human ESCs and fibroblasts reveals boundary localization to nucleosome-depleted regulatory elements and promoter marks, supporting similar results in yeast and mouse lines (Bonev et al., 2017; Hsieh et al., 2015, 2016). In particular, RAD21, CTCF, YY1, and ZNF143 exhibit enrichment at strong boundaries; however, boundaries are noted to be heterogeneous as some or all architectural factors and promoter marks do not occur at many boundaries (Krietenstein et al., 2020). Boundaries depleted of CTCF, YY1, and promoter marks were designated as weak boundaries; in addition to DNase I and GRO-seq signal, ChIP-seq analysis identified ASH2L, H3K4me3, SP1, CHD7, KDM1A, and HDAC2 as top features enriched at these boundaries. Following the role of RNA Pol II, Hsieh et al. also find evidence to support active transcription-mediated genome folding for the maintenance of E–P & P–P domains, thus mechanistically bridging form and function. Not only does gene compaction positively correlate with mammalian transcriptional activity (in contrast to findings in yeast (Hsieh et al., 2015)), but inhibition of Pol II significantly reduces the intensity of E–P and P–P stripes without affecting higher-order chromatin organization (Hsieh et al., 2020). Taking a polymer modeling-driven perspective in analyzing the nucleosomal interactome, both Hsieh et al. and Krietenstein et al. find evidence for nucleosomal clustering in clutches of ~3–10 nucleosomes locally interacting in trinucleosome or tetranucleosome zig-zag motifs. Importantly, both papers emphasize the heterogeneity of TADs and finer scales of organization, with Krietenstein et al. postulating loop transience governed by the extrusion complex’s interaction with loop anchors of varying strength.

**FIGURE 4:**
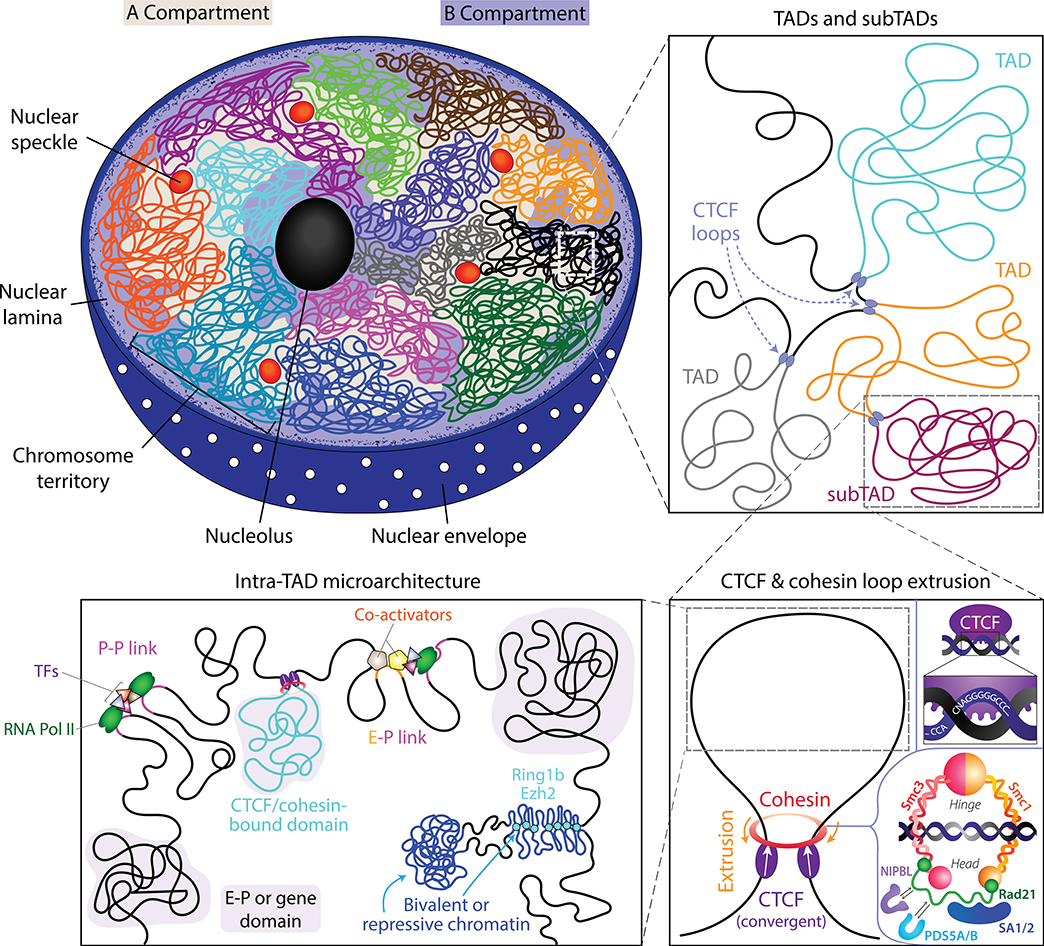
Current model for mammalian 3D genomic organization. Features of nuclear architecture are shown across scales of organization. (Top left panel) Chromosomes, hundreds of Mb in length, occupy distinct territories within the nucleus. Compartments up to of tens of Mb in length distinguish preferentially self-interacting domains of more transcriptionally active DNA (Compartment A) from less active DNA (Compartment B) and may interact across chromosomes. Nuclear bodies further define large-scale hubs of spatial organization, with denser, less active DNA clustering along the nuclear periphery and around the nucleolus and more accessible and active DNA clustering around nuclear speckles. (Top right panel) Topologically associating domains (TADs) arise at and below the Mb scale within each chromosome, with subTADs nesting within larger parent TADs. CTCF loops are identified by CTCF anchors at the point of contact at the base of a loop. (Bottom right panel) Loop formation is primarily driven by cohesin-mediated loop extrusion, which is halted by convergent CTCF sites. CTCF’s binding motif and components of the cohesin complex are shown. (Bottom left panel) Microarchitecture on the scale of hundreds of bp up to tens of kb consists of a diverse array of P–P and E–P linkages, small CTCF- and cohesin-mediated domains, bundles of repressive chromatin, and other gene domains. This panel is largely inspired by fig. 7f of Hsieh et al. (2020)

Collectively, these works revise our current model for fine-scale chromatin architecture within TADs and thus expand our understanding of different forces spanning the different scales of genome architecture. In addition to CTCF and cohesin, the primary determinants of TAD formation, Micro-C highlights the contributions of many other co-factors that affect fine-scale boundaries and organization below the typical scale of TADs. E–P or P–P stripes separate the intra-loop space into distinct domains, perhaps driven by active transcription and the convergence of transcription factors and co-activators. Polycomb proteins and local histone methylation create tightly packed pockets of repressive or bivalent chromatin contacts within larger euchromatic domains. And a repertoire of proteins contributes to this local folding by influencing both the strengths and locations of domain boundaries. Viewed through the collective lens of the field of genome organization, Micro-C’s ability to probe small-scale architecture thus offers a powerful tool for disentangling the biological complexity underlying folding. An updated model of nuclear architecture inspired by insights from Micro-C is shown in Figure 4.

## 5 CONCLUDING REMARKS & FUTURE PERSPECTIVES

In this paper, we review the development of chromosome conformation capture technologies, the biological mechanisms underpinning observed organizational features, and Micro-C’s contributions at the frontier of our understanding of nuclear architecture. Despite being a young field, chromosome conformation capture has rapidly deepened our understanding of the 3D genome and its influence on gene expression. Rapid innovation has spawned dozens of 3C- derived methods capable of extracting unique insights about chromosomal organization, answering some questions while also raising more. The conformation capture methods discussed here are by no means exhaustive; nevertheless, they are meant to reflect some of the major advancements in the field. The latest such advancement, Micro-C, brings genome-wide mapping of the interactome to the sub-kilobase scale, ushering in analysis of chromatin architecture at an unprecedented level. As the field transitions from an observational grounding to a functional one, Micro-C carries great promise for bridging genome structure to genome function.

The first applications of Micro-C in mammalian cells begin to disentangle mechanisms underlying loop formation (Hansen et al., 2019), underscore the heterogeneity of genomic organization, and identify a host of architectural proteins associated with different classes of fine-scale boundaries (Hsieh et al., 2020; Krietenstein et al., 2020). The mechanism by which the identified factors govern fine-scale boundaries remains unknown, as does the role of loop extrusion in CTCF-negative E–P and P–P domains. One possibility is that these architectural proteins are themselves capable of momentarily stalling cohesin’s procession, perhaps to a lesser degree than CTCF. Another is that E–P and P–P domains are formed independently of loop extrusion by mechanisms that remain to be elucidated. In addition to the causal roles of these factors in shaping organization, the functional relevance of E–P and P–P domains has not been characterized. Uncertainty remains as to whether these fine-scale structures themselves regulate transcription, or whether they are a by-product resulting from the process of transcription itself. Depletion and perturbation Micro-C experiments in the near future will likely shed light on both the mechanistic and functional implications of the observed fine-scale domains (Hsieh et al., 2020; Krietenstein et al., 2020).

Although the application of Micro-C in mammalian cells has yielded many novel insights, the technique is inherently limited in its capacity to address some outstanding questions and should therefore constitute one tool amidst a wider conformation capture toolkit. Adaptation of Micro-C to other Hi-C based techniques (e.g., in situ Micro-C, single-cell Micro-C, Capture-Micro-C) should further improve Micro-C’s signal-to-noise ratio and address cell-to-cell or region-specific variability in fine genomic architecture. Furthermore, like most 3C-based technologies, Micro-C is reliant upon proximity ligation and is limited to probing primarily pairwise contacts. For instance, with hundreds of known protein interactions with the promoter and enhancer regions demarcating fine-scale boundaries, it is possible that multi-way complex interactions at play in E–P and P–P stripes are better captured by non-3C-based methods.

Beyond chromosome conformation capture technologies, techniques including cryo-EM, fixed-cell microscopy, live-cell imaging, and polymer modeling will be instrumental for disentangling the mysteries of the 3D genome. The elucidation of the roles of CTCF and cohesin, the loop extrusion model, and the different forces at play across scales of organization have been foundational to the greatest breakthroughs in understanding chromosome conformation; however, many missing pieces in each of these stories remain to be found. Although significant biochemical evidence points toward cohesin-driven loop formation, the structure and molecular mechanism of the extrusion complex has not yet been captured. The biochemical mechanism explaining the convergent rule for CTCF anchoring of cohesin extrusion is still unclear, as are the forces contributing to compartmentalization. And even as the field has shifted away from a static view of TADs and other features of nuclear architecture, active loop formation and dynamics have not yet been observed in vivo. Finally, the extent to which genome organization is instructive for regulating transcription as opposed to a downstream consequence from the act of transcription itself remains an urgent but poorly understood question. Thus, a diverse repertoire of methods will be necessary to tackle looming questions in chromatin organization in coming years.

## BOX 1 THE NUCLEAR ARCHITECTʹS DICTIONARY

As the 3D genome field has grown rapidly, so too have the myriad of terms used to describe different features of nuclear architecture. Unfortunately, there is currently no clear consensus on terminology, and generally accepted and precise definitions are lacking for most terms. This is partly due to the complexity of nuclear organization and due to the numerous mechanisms and forces acting simultaneously upon a given locus or domain. For a more comprehensive discussion of nomenclature we refer the reader to reviews discussing terminology in depth (Beagan & Phillips-Cremins, 2020; Rowley & Corces, 2018).

For the purposes of this review, we use the following terminology. We refer to *contact domains* as an umbrella term describing any domain visible in contact maps as a square or triangle, thus corresponding to regions of elevated chromatin interactions. Contact domains can be subdivided into two major categories— compartment domains and Topologically Associating Domains (TADs). *Compartment domains*, comprised of self-associated A or B compartmentalized chromatin, manifest in a checkerboard manner across contact maps and are typically assigned using eigenvector decomposition. *TADs*, on the other hand, are local domains that manifest as triangles rising off of the contact map diagonal and are usually called based on an insulation score or directionality index (de Wit, 2020). Mechanistically, TADs are thought to be formed by loop extrusion, whereas compartmentalization is less well understood but may arise due to block copolymer microphase separation. The mechanisms responsible for forming compartments and TADs likely act upon all genomic segments, making them challenging to distinguish. Moreover, nested compartments and TADs can be further divided into *subcompartments* and *subTADs*. Contact lines extending past the edges of a TAD are referred to as *stripes, flames, flares,* or *tracks.* If the corner of a TAD is a distinct point of interaction on the contact map, then it is referred to as a *dot* or *corner peak.* These dots and corner peaks are biologically interpreted as chromatin loops. Thus, contact domains and TADs with clear corner peaks are often referred to as *loop domains*. The subset of corner peaks anchored by bound CTCF sites (e.g., via ChIP-seq analysis) are called *CTCF loops*. Finally, dots and corner peaks between enhancers and promoters are referred to as *enhancer-promoter (E-P), enhancer-enhancer (E-E),* and *promoter-promoter (P-P) interactions, loops,* or *links.*

Finally, we note that features such as compartments, TADs, and CTCF loops are generally population-level terms that are visible in contact maps after averaging across millions of heterogenous cells. Different terminology is sometimes used for domains observed using single-cell methodologies that may be present in single cells, but sufficiently rare to not be clearly visible in population-averaged contact maps. For instance, “TAD-like domains” of contact that manifest in single-cell data may be a snapshot of the intermediary formation of a domain observed in population-level data (Bintu et al., 2018). We also note that different architectural terms may be used to describe organizational features in different organisms and that the same term may hold different denotations in different organisms (Szabo, Bantignies, & Cavalli, 2019).

For the purposes of this review, we refer to TADs as local organizational domains formed primarily by loop extrusion. Our use of the term is not intended to omit the validity of other applicable terms, but is done for the sake of consistency throughout this review.
